# Requiring Pollutant Discharge Permits for Pesticide Applications that Deposit Residues in Surface Waters

**DOI:** 10.3390/ijerph110504978

**Published:** 2014-05-08

**Authors:** Terence Centner, Nicholas Eberhart

**Affiliations:** Department of Agricultural and Applied Economics, College of Agricultural and Environmental Sciences, The University of Georgia, Athens, GA 30602, USA; E-Mail: eberhart@uga.edu

**Keywords:** pesticides, registration, water quality, pollutant discharge permits, pesticide residues

## Abstract

Agricultural producers and public health authorities apply pesticides to control pests that damage crops and carry diseases. Due to the toxic nature of most pesticides, they are regulated by governments. Regulatory provisions require pesticides to be registered and restrictions operate to safeguard human health and the environment. Yet pesticides used near surface waters pose dangers to non-target species and drinking water supplies leading some governments to regulate discharges of pesticides under pollution discharge permits. The dual registration and discharge permitting provisions are burdensome. In the United States, agricultural interest groups are advancing new legislation that would exempt pesticide residues from water permitting requirements. An analysis of the dangers posed by pesticide residues in drinking water leads to a conclusion that both pesticide registration and pollutant discharge permitting provisions are needed to protect human health and aquatic species.

## 1. Introduction

Food production relies heavily on the use of pesticides for growing crops and raising animals. Data suggest that approximately 2.36 billion kg of pesticides are used each year in the World [1]. In the United States, agricultural uses of pesticides account for nearly 80% of the 0.5 billion kg of pesticides applied [1]. Many pesticides are carcinogens and their widespread usage raises concern about the possibility of negative impacts on people and the environment [2,3]. Given the dangers posed by pesticides, countries have adopted detailed regulations governing their production and use [4,5,6,7,8]. In the United States, Congress enacted the Federal Insecticide, Fungicide, and Rodenticide Act to oversee the manufacture, distribution, and use of [9]. Developed countries have designated a governmental agency to oversee the registration, labeling, and use of pesticides. Countries without adequate national legislation on pesticides may subscribe to the International Code of Conduct on the Distribution and Use of Pesticides adopted by the Food and Agricultural Organization of the United Nations [10].

A contemporary issue is whether the use of pesticides accompanied by the deposition of residues into surface waters should be regulated by water quality control provisions. These deposits may occur during aerial applications of pesticides to control agricultural and forest pests as well as disease-carrying mosquitoes [11]. Requiring persons who discharge pollutants into waters to file permits delineating the minimization of pollutant discharges is important for safeguarding water quality and public health [12,13,14,15]. Yet, in 2006 the U.S. federal government concluded that a pollutant discharge permit was not required [16]. EPA felt that pesticides were neither “chemical waste” nor “biological materials” so were not subject to the permitting provisions [16].

This federal regulation was overturned in the *National Cotton Council of America v. U.S. EPA* lawsuit [17]. In response to the court’s ruling, EPA developed a “Pesticide General Permit” that would facilitate the issuance of pollutant discharge permits for applications of pesticides depositing residues into surface waters [18]. Agricultural interest groups seek to enact new legislation to reverse the permit requirement.

Jurisprudence that recognizes pesticide residues deposited in water as pollutants requiring a pollutant discharge permit is accompanied by concerns about the costs of the requirement. Interest groups representing agricultural and public health applicators object to the requirement of a pollutant discharge permit because securing a permit is time consuming and costly. In response to these arguments, interest groups in the United States have proposed legislation that says the pollutant discharge permitting provisions for water quality do not apply for applications of registered pesticides. Under the proposed legislation, applications of pesticides to eliminate pests carrying diseases and damaging crops could proceed without a pollutant discharge permit despite deposits of pesticide residues in surface waters. Simultaneously, other evidence suggests that deposits of pesticides into waters contribute to increased health costs [14]. To determine whether pollutant discharge permitting provisions are needed, the registration and permitting provisions are analyzed. The analysis discloses that they address different problems and the pollutant discharge permitting provisions are needed to protect drinking water supplies.

## 2. Regulatory Oversight

### 2.1. Registration

Countries regulate the manufacture, distribution, sale, and use of pesticides with an objective of protecting public health and the environment [3]. Pesticide registration is a scientific and administrative procedure through which a governmental agency examines the ingredients of the pesticide, the particular site or crop on which it is to be used, the amount and frequency of its use, and storage and disposal practices [19,20]. Applications delineate the intended target crop, claims of pesticide effects, and scientific data supporting the claims. Separate registration is required for different crops, pests, and dosage levels [20,21]. While the initial provisions of a registration program are primarily implemented in order to monitor the efficacy of pesticides, provisions may also consider environmental and health concerns.

Pesticide registration regulations also contain limitations that deny registration to highly toxic pesticides or pesticides with unknown toxicity. In the United States, only pesticides performing their intended function “without unreasonable adverse effects on the environment” can be registered [9]. Unreasonable adverse effects on the environment consider economic, social, and environmental costs and benefits of the use of a pesticide. By employing a cost-benefit analysis for analyzing the risks that accompany the use of pesticides, registration is denied to those that pose too many dangers to humans or the environment. The cost-benefit analysis offers a quantitative approach to addressing risk, although controversies exist on how to evaluate ecological values, community values, and normative considerations [22].

### 2.2. Discharge Permits

While pesticide registration generally considers water quality in approving the registration of a pesticide, registration provisions are quite different from the provisions incorporated in discharge permits (Table 1). This means that registration does not offer the same degree of protection to public drinking water supplies as is offered by discharge permits. Registration fails to address actual discharges of pesticides into surface waters so cannot preclude unhealthy concentrations of pesticides in drinking water supplies [14]. Moreover, registration does not assign liability for negligent pesticide applications. Thus a government may decide that a pollution discharge permit is needed for pesticide applications depositing pesticide residues in surface waters. A permitting program incorporates responsibilities for pesticide applicators that operate to reduce the discharges of pollutants into surface waters.

**Table 1:** Contrasting U.S. registration with discharge permits.

Environmental Issue	Registration	Discharge Permits
Deposits of residues in surface wasters	No control over specific applications that cause residues to be deposited in surface waters	Controls over applications to minimize amounts of toxics entering surface waters
Managing residues in runoff	Dosage levels for individual crops	Dosage levels that consider levels of toxic substances in waters
Weighing harm	Cost-benefit analysis of adverse effects on the general environment	Consideration of expected harm from toxics deposited in surface water
Consideration of ecology	Unclear consideration of ecological values	Consideration of harm from concentrations and accumulations
Regulatory oversight	Enforcement by the federal and state governments	Enforcement by governments and by citizen lawsuits

Pesticides in waters used for human consumption present worrisome public health concerns [23]. Pesticides are toxic chemicals designed to control and eliminate pests and pose significant health risks to humans or other non-target organisms. While the differing exposure periods, toxicities, and contamination levels complicate the study of exact human health effects of pesticides, a European study of pesticide toxicity showed 81 pesticides out of 276 legally marketed active substances approved for use in Europe were toxic substances with possible adverse effects on human health [20]. Therefore, applications of pesticides that result in residues in drinking water supplies can negatively affect human health.

Research on potential health problems associated with pesticide use identifies concerns about their adverse effects on humans. In some cases, the dangers posed by pesticide use have been recognized. For example, endosulfan is an endocrine disruptor that was added to the list of persistent organic pollutants of the Stockholm Convention [24]. Pesticides affect oxidative stress that may be associated with an increased risk of cancer [25]. Considerable research links increased prostate cancer with the use of pesticides [26]. In analyzing four insecticides, researchers found that fonofos, malathion, terbufos, and aldrin are associated with the risk of aggressive prostate cancer [27]. Other research suggests that pesticides contribute to the pathogenesis of Parkinson’s disease [14].

Most pesticides found in water are at low concentrations so do not pose immediate health effects. However, they can have adverse chronic effects due to long-term exposure [28,29]. Drinking water is thought to be an important source of exposure of organophosphate pesticides that harm brains in children [30]. Pesticides at low doses may exert neurotoxic effects [31]. Research suggests that exposure to pesticides can cause the dysregulation of immune functions that could contribute to immunodeficiency, tumorigenesis, allergies, and autoimmunity [32]. Chronic effects of pesticides in water supplies are difficult to observe directly due to their delayed effects.

These data suggest that because pesticide registration does not address deposits and accumulations of pesticides in drinking water supplies that may cause cancer and increase health maladies, the regulation of pollutant discharges is needed. Unauthorized deposits of pesticides in surface water are discharges of pollutants. Whenever a pesticide applicator places pesticide residues in surface waters without authorization, it presents risks of health and environmental damages. Governments may want to manage these risks through a pollutant discharge permitting system, and may seek to have citizens help with the enforcement of permitting provisions.

### 2.3. National Primary Drinking Water Regulations

Under federal law, National Primary Drinking Water Regulations establish maximum contaminant levels and treatment techniques that govern public water supplies [33]. These requirements are intended to protect persons from too high of concentrations of known contaminants. While this should protect persons ingesting water from public water sources, they do not cover all contingencies. Statistics by the EPA reported that 3% of public water systems had experienced at least one health-based violation affecting 6% of the population [34].

The public requirements are not applicable to private wells. Water from many private wells remains untested. A Pennsylvania study found that 30% of private well owners had never tested their water [35]. A study in selected New England counties suggested that 30% of population using private wells had arsenic concentrations that exceeded recommended concentrations [36]. When water testing is performed, it often looks at individual chemicals so fails to account for compounds [37]. Testing also fails to consider cumulative risk assessments [37].

## 3. Pollution Examples from the United States

### 3.1. Litigated Cases

Unwanted registered pesticides have entered surface waters and caused damages. In the state of Oregon, a lawsuit was filed against an irrigation district for its deposits of the registered aquatic herbicide Magnacide H (acrolein) in canals that later contaminated surface waters downstream [38]. The application of this pesticide resulted in contamination of a local creek and the death of fish in downstream surface waters. The defendant pesticide applicator argued that a point-source pollutant discharge permit was not needed as such was not indicated on the pesticide's label. The court concluded that pesticide registration and pollutant discharge permitting involve different issues and that the registration of the pesticide did not obviate the need for a discharge permit.

A second lawsuit challenged a U.S. federal regulation that said no point-source pollutant discharge permit was needed for most applications of registered pesticides that resulted in deposits of pesticide residues in surface waters [17]. Environmental groups felt the regulation inadequately regulated pesticide applications that placed residues in water. The court ruled that federal law required a pollutant discharge permit for chemical waste and biological materials including pesticides being placed in surface waters. Given the ruling by the court, the federal government developed a “Pesticide General Permit” that would facilitate the issuance of pollutant discharge permits for applications of pesticides depositing residues into surface waters [18].

### 3.2. Need for Protection of the Environment and Human Health

Pesticides entering water bodies pose threats to the ecological integrity of surface waters [39]. Research suggests that pesticides in surface waters have a negative impact on a high number of non-target species, with macroinvertebrate species especially vulnerable [40,41]. Amphibians and fish may also be adversely affected [42,43].

Research by the United States Geological Survey suggests that over 9% of stream water in agricultural areas have pesticide concentrations greater than human-health benchmarks, and 6% of the streams in urban areas [37]. Other research taking surface water samples from three California agricultural regions showed a toxicity benchmark was exceeded in 19% of the samples [44]. Moreover, because water sampling often fails to account for chemical compounds, additional unknown toxicities may exist [37]. To adequately protect the environment and human health, more might be done to reduce pesticides residues entering surface waters.

### 3.3. State Permitting Provisions

Some U.S. state governments have proceeded to adopt permitting provisions to regulate pesticide deposits in surface waters. Oregon adopted a state Pesticide General Permit in 2011 covering applications of biological and chemical pesticides in, over, or near water. The Oregon permit regulates pesticide applications used to control mosquitoes and other flying insects, weeds and algae, nuisance animals, forest canopy pests, and area-wide pests [45]. Oregon has also proposed a general permit for pesticide use in irrigation system boundaries [46]. Washington state adopted an Aquatic Mosquito Control General Permit in 2010 [47] and a Noxious Weed Control NPDES General Permit in 2012 [48]. Other states are developing general permit programs to address pesticide residues entering surface waters [49]. These regulations require permits where pesticides or other products may enter surface waters.

## 4. Proposed U.S. Federal Legislation

Interest groups opposing the requirement of pollutant discharge permits for pesticides entering surface waters have asked U.S. congressional representatives to introduce federal legislation that would amend federal regulations to exempt pesticides from pollutant discharge permitting requirements [50]. The most familiar proposal would amend both the federal pesticide registration and water quality laws to preclude requirements of pollutant discharge permits for pesticide discharges [51]. The proposal would allow applications of pesticides for agricultural, forestry, and mosquito control purposes that place toxic chemicals in drinking water supplies without oversight of toxicity levels. It would also preclude U.S. state governments from requiring discharge permits.

In eliminating pollutant disposal permits, the proposal would markedly reduce the health and environmental protections currently provided by federal and state water quality regulations. Four changes to U.S. federal law can be discerned: (1) obviating permitting costs; (2) allowing accumulations of pesticides; (3) reducing states’ rights; and (4) reducing enforcement activities. These proposed changes offer governments options in their regulation of risks accompanying the use of pesticides.

### 4.1. Obviating Pollutant Discharge Permitting Costs

Agricultural interest groups and municipal governments applying pesticides to control pests are concerned about the costs that accompany pollutant discharge permits [52]. The Pesticide General Permit adopted by the U.S. government in 2011 requires regulation of 5.6 million pesticide applications by 365,000 new permit holders [50]. Agricultural and forestry interest groups argue that the permitting processes for these industries will negatively impact their productivity and profitability [52].

However, the requirement of pollutant discharge permits under a Pesticide General Permit for pesticide discharges into surface waters does not affect most agricultural producers. Only pesticide applications that discharge pollutants into surface waters are regulated. Because most farmers applying pesticides to their crops in fields, groves, orchards, and vineyards do not discharge pesticides into waters, they do not need a pollutant discharge permit. This means that the pollutant discharge permitting requirements for applicators placing pesticide residues in waters would have no impact on the vast majority of agricultural producers.

### 4.2. Allowing Accumulations of Pesticides

The legislative proposal to preclude requirements of pollutant discharge permits for pesticide discharges would remove governmental controls that oversee risks posed by accumulations of pesticide deposits in surface waters. Considerable evidence shows public health risks in situations where pesticide residues accumulate in drinking water sources [42]. The cost-benefit analysis employed for registering pesticides under U.S. pesticide registration law allows pesticides to be released without an evaluation of concentrations of substances at specific locations.

The legislative proposal changes federal law to allow pesticide residues inimical to public health to be deposited in surface waters. Because multiple applications of pesticides may cause pesticide residues to accumulate in a waterbody [42], unacceptable concentrations of toxins in drinking water that impair public health may occur from registered pesticides. An exemption from pollutant discharge permitting provisions would allow applicators to deposit pesticides in drinking water supplies even when concentrations of residues are at levels known to cause cancer or other health maladies. This would occur because FIFRA registration does not consider accumulations at specific locations.

### 4.3. Reducing Rights of State Governments

Another issue involves the nullification of U.S. states’ rights by the provisions delineated in the federal legislative proposal. It specifically precludes states from requiring permits for pesticides entering surface waters. This is the antithesis of the current policy embedded in federal law under which U.S. states can offer citizens protection against pollutants and take action to foster cleaner water resources for tourism and recreational pursuits [53,54].

Most U.S. states have chosen to self-administer their pollutant discharge permitting programs so that they have more control over in-state water resources and economic development [55]. The proposal would preclude U.S. states from effectively regulating pollution and protecting people against pollutants from other states [56]. The proposal would also preclude states from innovating and experimenting in attempts to address specialized needs or effectuate state objectives [21].

### 4.4. Reduced Enforcement Opportunities

The proposed legislation would reduce enforcement opportunities for challenging unauthorized applications of pesticides. Although federal pesticide registration provisions require compliance with label warnings, it lacks effective control mechanisms to ensure compliance [57]. There is no requirement in federal pesticide law to consider locally-based water quality standards or the cumulative toxic effects of separately applied pesticides on drinking water quality [57]. Lawsuits concerning fish kills and mosquito control programs identify situations where applications of registered pesticides may harm human populations.

A reduction in enforcement opportunities would result from the elimination of the ability of citizens to bring legal action under a “citizen suit” [58]. The citizen suit provisions allow individuals to act as “private attorneys general” to bring lawsuits enforcing pollution standards [9]. Citizen suit provisions were crafted because governments lack the resources to monitor polluters and cannot always be effective in enforcing the law. Citizen suit provisions also act to motivate government enforcement and abatement proceedings. The proposed legislation to eliminate requirements for pollutant discharge permits for pesticide discharges into surface waters would preclude citizens from taking actions against applicators who deposit pesticides in water.

These reduced enforcement opportunities would be in addition to the inability of governments to monitor existing pesticide applications. The lack of monitoring is significant given the practices that may be used by applicators causing unnecessary contamination. An applicator may use faulty equipment that causes unnecessary discharges into waters. To save time, an applicator may fail to correctly calibrate equipment, set the spray boom too high above the crop, or apply too fine a mist causing excessive discharges to enter surface waters. Other application problems include applying pesticides when it is too windy, spraying too close to surface waters, and applying when the weather is too warm and humid causing excessive drift. A permitting system is helpful because it requires applicators to delineate management practices for minimizing drift. Thereby, permit applications provide applicators an opportunity to contemplate their responsibilities for using pesticides near waters. Applicators are reminded of practices that can reduce the potential of pesticides entering surface waters.

## 5. Conclusions

Many pesticides are dangerous carcinogens. Countries have enacted pesticide registration laws to regulate the use of pesticides through registration programs based on generalized cost-benefit analyses using data submitted by the registrant. Registration provisions are not intended to preempt other governmental directives needed to protect people and the environment from harm. Pesticides deposited into surface water bodies pose hazards to the environment and public health. Thus, governments may decide to regulate deposits into surface waters under a pollutant discharge permitting program.

In the United States, agricultural interest groups have proposed a federal law that would exempt pesticides from pollutant discharge permitting requirements and preclude U.S. state governments from adopting requirements deemed necessary to protect drinking water supplies and aquatic species. An analysis of federal registration and water quality laws, as well as scientific research on human health concerns related to pesticide accumulations in drinking water, supports a conclusion that deposits of pesticides in surface waters need to be regulated by a pollutant discharge permitting program. Pesticide registration only considers overall damages and does not reveal damages associated with pesticide concentrations and accumulations in surface waters. Registration provides no monitoring of pesticide applications near surface waters and no liability mechanism for wrongful pesticide applications. Unregulated concentrations of pesticides in waters used for drinking, irrigating crops, and the production of aquatic species for food may adversely affect humans.

Given growing scientific evidence of health problems related to pesticides in drinking water, governments should be increasing their efforts to reduce amounts of pesticides in surface waters to control health costs. The European Union Parliament recognized the need to reduce the adverse effects of pesticides in Directive 2009/128/EC [4]. Member states are to prohibit aerial spraying except for special cases, and when employed, aerial spraying must use the best available technology to reduce spray drift. Buffer zones are suggested near aquatic environments. Pesticide applicators in the United States need to minimize harmful discharges of pollutants in surface waters. Because pesticide registration fails to embody this objective, pollutant discharge permits are needed to protect persons from harmful pesticide residues.
